# New Functions of APC/C Ubiquitin Ligase in the Nervous System and Its Role in Alzheimer’s Disease

**DOI:** 10.3390/ijms18051057

**Published:** 2017-05-14

**Authors:** Tanja Fuchsberger, Ana Lloret, Jose Viña

**Affiliations:** Department of Physiology, Faculty of Medicine, University of Valencia, INCLIVA Avda. Blasco Ibañez 15, 46010 Valencia, Spain; tania.fuchsberger@uv.es (T.F.); jose.vina@uv.es (J.V.)

**Keywords:** ubiquitin ligase, neurodegeneration, excitotoxicity, oxidative stress

## Abstract

The E3 ubiquitin ligase Anaphase Promoting Complex/Cyclosome (APC/C) regulates important processes in cells, such as the cell cycle, by targeting a set of substrates for degradation. In the last decade, APC/C has been related to several major functions in the nervous system, including axon guidance, synaptic plasticity, neurogenesis, and neuronal survival. Interestingly, some of the identified APC/C substrates have been related to neurodegenerative diseases. There is an accumulation of some degradation targets of APC/C in Alzheimer’s disease (AD) brains, which suggests a dysregulation of the protein complex in the disorder. Moreover, recently evidence has been provided for an inactivation of APC/C in AD. It has been shown that oligomers of the AD-related peptide, Aβ, induce degradation of the APC/C activator subunit cdh1, in vitro in neurons in culture and in vivo in the mouse hippocampus. Furthermore, in the AD mouse model APP/PS1, lower cdh1 levels were observed in pyramidal neurons in CA1 when compared to age-matched wildtype mice. In this review, we provide a complete list of APC/C substrates that are involved in the nervous system and we discuss their functions. We also summarize recent studies that show neurobiological effects in cdh1 knockout mouse models. Finally, we discuss the role of APC/C in the pathophysiology of AD.

## 1. Introduction

The ubiquitin-proteasome system allows dynamic regulation of cell functions by targeting proteins for degradation. Thereby, E3 ubiquitin ligases provide substrate specificity for the conjugation of ubiquitin. In the brain, ubiquitin ligases have a critical role in the regulation of neuronal morphology and connectivity. They are localized within distinct subcellular compartments in neurons, such as the nucleus, axons and dendrite cytoskeleton, synapses, centrosome, and Golgi apparatus, allowing for the ubiquitination of local substrates [1]. 

Anaphase Promoting Complex/Cyclosome (APC/C) belongs to the cullin-RING ubiquitin ligases, which represent the largest known class of E3 ubiquitin ligases [2]. It is a multimeric protein complex that consists of 15 different proteins assembled to 20 subunits. The activity of APC/C strictly depends on regulatory coactivator subunits, which promote the interaction of APC/C and the substrate and stimulate the catalytic reaction [3]. They are termed cell-division cycle protein 20 (cdc20) and cdc20 homologue-1 (cdh1) [4]. The coactivators recognize conserved destruction motifs, such as the D box (RxxLxxxxN/D/E), KEN box (KENxxxN), and Cry box (CRYxPS) [5,6,7] and recruit the substrates to the APC/C. 

Besides the critical role of the timed activation of APC-Cdc20 and APC/C-Cdh1 in the cell cycle, APC/C is involved in many other essential processes including genomic integrity, metabolism, as well as functions in development and in the nervous system [8,9,10]. 

First indications for a role of APC/C in the nervous system were provided by mRNA expression data of the APC subunits in neurons. Gieffers et al. (1999) reported that some APC/C subunits and cdh1 were ubiquitously expressed in fully differentiated cultured rat hippocampal neurons [11], which initiated the search for functions of APC/C-Cdh1 in the nervous system. Since then, several fundamental processes, such as axonal growth and patterning [12,13], neuronal cell cycle exit [14], neuronal differentiation [15] and neurogenesis [10], regulation of homeostatic plasticity [16], long-term potentiation [17], and long-term depression [18], have been shown to be regulated by APC/C-Cdh1. Furthermore, it has been reported that APC/C-Cdc20 is involved in the control of dendrite morphogenesis in post-mitotic neurons [19] and presynaptic differentiation [20]. To date, 15 substrates of APC/C have been identified that have a direct implication on the nervous system (see Table 1). 

Interestingly, impaired function of APC/C and accumulation of its substrates have been related to neurodegenerative diseases, as it was shown to be involved in excitotoxicity [21,22], oxidative stress [23], and ectopic cell-cycle re-entry [24,25,26,27]. Moreover, it has been reported recently that Aβ causes a proteasome-dependent degradation of cdh1 [21], suggesting its involvement in Alzheimer’s disease (AD).

## 2. APC/C-Cdh1 and Axon Growth in the Cerebellar Cortex

In the adult brain, axon growth is limited by intrinsic and extrinsic inhibition, which is essential for a correct neuronal architecture. Konishi et al. (2004) showed that APC/C controls axonal growth and patterning in mammalian neurons [12]. The acute knockdown of cdh1 in primary cerebellar granule neurons by RNA interference induces a change in the morphology of neuronal processes. By measuring the growth of dendrites and axons (identified by the somato-dendritic marker MAP2 and the axonal marker Tau), it was observed that cdh1 knockdown caused a substantial increase in growth rate and a significant elongation of the axonal length, but not of dendrites. Moreover, the depletion of the subunit APC11 or the inhibition of APC/C by Emi1 in granule neurons also induced axonal growth. In addition, this was tested in vivo by subjecting sh-cdh1 plasmid to the cerebellar cortex of P6 rat pups. Shcdh1-expressing granule neurons were drastically altered and several axons grew abnormally off a regular track in parallel fibers. Furthermore, cdh1 knockdown was shown to override the inhibitory effect of adult rat myelin on axon growth [12]. 

Stegmüller et al. (2006) reported a mechanism by which the ubiquitin ligase APC/C-Cdh1 controls axonal morphogenesis [13]. They showed that APC/C-Cdh1 operates in the nucleus for inhibition of axonal growth by targeting the transcriptional corepressor Ski-related novel protein N (SnoN) for ubiquitin-dependent proteasomal degradation. The knockdown of SnoN in primary cerebellar granule neurons reduced the growth of axons, whereas high protein levels of SnoN stimulated it. This was confirmed in vivo by knockdown of SnoN in the developing rat cerebellum, which caused an inhibition of the elongation of granule neuron parallel fibers. Additionally, it was shown that the APC/C-Cdh1/SnoN pathway is co-regulated by TGFβ/Smad2. Smad2 associates with SnoN in neurons and recruits APC/C-Cdh1 to its substrate. This is promoted by TGFβ, which induces the translocation of Smad2 to the nucleus, where it interacts with cdh1. Inhibition of TGFβ inactivates Smad2 signaling, which causes an increase in SnoN, and axonal growth is thereby stimulated [13,20]. 

Furthermore, another APC/C-Cdh1 substrate, the inhibitor of DNA binding 2 (Id2) protein, is involved in axon growth regulation [14]. In the developing nervous system, Id2 inhibits neurogenic basic helix-loop-helix transcription factors, which enhances cell proliferation and promotes tumor progression. In neurons, Id2 is degraded by APC/C-Cdh1 when the cells enter a quiescent state, which allows the accumulation of axon growth inhibitors. This is mediated by the accumulation of helix-loop-helix transcription, which induces the expression of genes with inhibitory effects on axonal growth, e.g., the Nogo receptor. It was shown that depletion of cdh1 stabilizes Id2, which leads to increased axon growth in cerebellar granule neurons [14]. 

**Figure 1:**
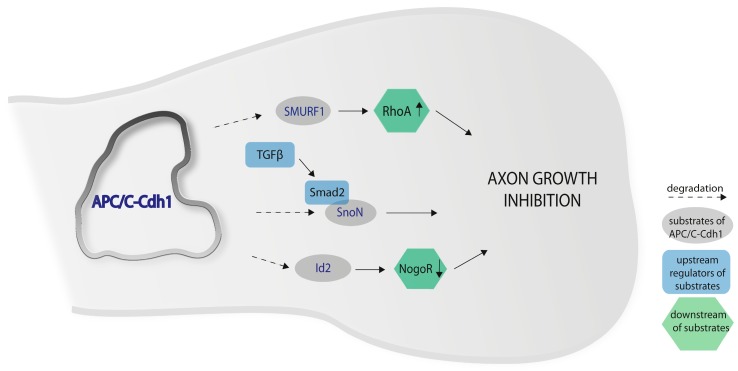
Anaphase Promoting Complex/Cyclosome regulates axon growth. APC/C-Cdh1 targets the ubiquitin ligase Smurf1 for degradation, which allows for the accumulation of RhoA, a potent regulator of axon growth. TGFβ/Smad2 signaling recruits APC/C-Cdh1 to its substrate SnoN, an axon growth stimulating protein. The destabilization of the transcription factor Id2 limits the expression of genes with inhibitory effect on axonal growth like the Nogo Receptor.

The E3 ubiquitin ligase Smurf1 is another APC/C-Cdh1 target, which is involved in axon growth regulation. Smurf 1 had previously been related to axon initiation and growth of neurons [38,39]. Knockdown of Smurf1 in cerebellar granule neurons causes a decrease in axonal length. In vivo knockdown of Smurf1 causes a disruption of neuronal migration in the developing cerebellar cortex. When Smurf1 is stabilized, it overcomes myelin-induced inhibition of axon growth. Furthermore, it was shown that a protein targeted by Smurf1, the small GTPase RhoA, has a crucial role in axon growth inhibition. It was shown that APC/C-Cdh1 acts upstream of the Smurf1/RhoA pathway in the control of axon growth and that this pathway acts in parallel to the previously identified substrates SnoN and Id2 [31] (Figure 1). 

## 3. APC/C-Cdh1 and Neurogenesis

APC/C-Cdh1 ubiquitin ligase activity is required for neurogenesis in vivo. In embryo-restricted cdh1 knockout mice, it was shown that the cell cycle exit is delayed in neural progenitor cells, causing replicative stress and p53-mediated apoptotic death. The phenotype of these mice exhibits increased length of the ventricular and the subventricular zone. Cortical neurons are decreased in number and a reduction of cortical size occurs [10]. These alterations are mediated by the accumulation of Skp2, an F-box protein that had previously been described as a substrate of APC/C-Cdh1 [40]. Under normal conditions, the degradation of Skp2 promotes the stabilization of p27, which is an inhibitor of cyclin-dependent kinases, and that results in cell cycle exit. Furthermore, it was shown that E2F3A, an APC/C-Cdh1 substrate, decreases steadily in differentiating neuroblastoma cells in an APC/C-Cdh1-dependent manner, which was accompanied by an increase in p27 and reduced levels in cyclin A [33].

The phenotype of these embryo-restricted cdh1 knockout mice resembles that of microcephaly. Interestingly, the Drosophila homolog of MCPH1 B, a protein encoded by a causative gene of autosomal recessive primary microcephaly, was identified as a substrate of APC/C-Cdh1 [37]. However, further research will be needed to elucidate a possible implication of APC/C dysfunction in microcephaly. 

Eguren et al. (2013) reported that mice, in which cdh1 was eliminated in the developing nervous system [41], suffered defects in neuronal progenitor cells, accumulation of cerebrospinal fluid in the brain cavities, and death. In cdh1-depleted neuronal progenitors, replicative stress induces p53-dependent apoptosis. The ablation of p53 prevents apoptosis, but not replicative stress, which results in the presence of damaged neurons in the adult brain. Summarized, this study shows that cdh1 is required during neurogenesis as it prevents replicative stress. 

Furthermore, it has been shown that casein kinase 1 (ck 1) δ is an APC/C-Cdh1 substrate and that it regulates neurogenesis in cerebellar granule cells [32]. The ck 1 family is highly conserved in eukaryotes and controls a broad spectrum of biological processes, e.g., circadian rhythms, signal transduction, apoptosis, and neurite outgrowth. It was shown that ck 1 δ is degraded by APC/C-Cdh1 in a D-box dependent manner and knockout of cdh1 in cerebellar granule cell progenitors (GCP’s) stabilizes ck 1 δ. APC/C-Cdh1 normally downregulates ck 1 δ during cell cycle exit and thereby controls the balance of proliferation and cell cycle stop in the developing brain. Overexpression of this kinase results in increased proliferation, while loss of ck 1 δ inhibits the expansion of GCP’s [32]. 

**Figure 2:**
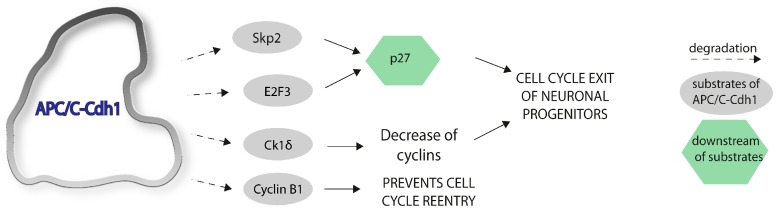
APC/C-Cdh1 regulates neurogenesis and cell cycle exit. Various substrates of APC/C-Cdh1 have been shown to be involved in the regulation of the cell cycle of neuronal progenitors: Skp2, E2F3A, Ck1 δ. Cyclin B1 downregulation is responsible for maintaining the cell cycle exit.

The downregulation of cyclin B1 is necessary for the maintenance of cell cycle exit of differentiated neurons, and this will be discussed in more detail in the following sections. The substrates of APC/C-Cdh1 involved in neurogenesis and cell cycle exit are summarized in Figure 2. 

## 4. APC/C-Cdh1 Is Involved in Synaptic Plasticity

In Drosophila, APC/C subunits and cdh1 are located at neuromuscular synapses. Loss of function of APC/C subunits leads to overgrowth of synaptic boutons. That is accompanied by altered synaptic transmission and leads to an increase in the amount of postsynaptic glutamate receptors. This was correlated to high levels of Liprin-α, a possible substrate of APC/C [28]. 

APC/C downregulates GLR1 levels, a non-NMDA glutamate receptor (GluR) in the nematode Caenorhabditis elegans. Mutations in APC/C subunits increased GLR1 in the ventral nerve cord, and the overexpression of ubiquitin reduces GLR1 in synapses. APC/C mutants have increased synaptic strength and that causes defects in locomotion. However, based on amino-acid sequence analysis, it does not seem likely that GLR1 is a direct target of degradation of APC/C, and the authors hypothesized that GLR1 might be associated to a scaffolding protein that is targeted for ubiquitination [42]. 

Moreover, a role of APC/C-Cdh1 in synaptic plasticity was shown in the mammalian brain in rodents. It is involved in the regulation of homeostatic plasticity, which is the counteraction on synaptic strength to unrestrained changes, maintaining neuronal output. This occurs when chronic elevation of synaptic activity reduces the amplitude of miniature excitatory postsynaptic currents (mEPSC). EphA4 belongs to the Eph receptor family, which is involved in the regulation of excitatory neurotransmission and plays a role in cytoskeleton remodeling. Elevated synaptic activity leads to increased phosphorylation of EphA4, which allows its association with APC/C-Cdh1. That causes GluR1, a subunit of AMPA receptors, to be targeted for degradation, resulting in an attenuation of the mEPSC amplitude. The depletion of cdh1 in neurons in vitro prevented the EphA4-dependent degradation of GluR1 [16].

### 4.1. APC/C-Cdh1 and Long-Term Potentiation

Li et al. (2008) showed that APC/C-Cdh1 is involved in learning and memory. In this study, cdh1-deficient mice, in which a gene-trap construct was inserted in intron 5 of cdh1, were analyzed. The lack of cdh1 causes early lethality in homozygous mice. In heterozygous mice, in which cdh1 was decreased by approximately 50%, late-phase long-term potentiation (LTP) in the hippocampus was impaired. Furthermore, defects in contextual fear-conditioning occurred in these animals [17].

In a conditional knockout model, in which cdh1 was specifically deleted in neurons at the onset of differentiation, a role of cdh1 in LTP was confirmed. Hippocampal slices from these cdh1 knockout mice displayed reduced late-phase LTP. Furthermore, they displayed impaired flexibility in behavioral tasks and reduced extinction of associative fear memory. However, in this model, spatial memory tasks were not affected [43]. 

The effects of post-developmental removal of cdh1 were tested by deleting cdh1 in adult mice from excitatory neurons in the forebrain. In coronal slices from the amygdala, late-phase LTP was impaired in the knockout mice compared to wild types when thalamic afferents were stimulated and recorded in the amygdala. After the LTP-inducing stimulation, the proteins Shank1, a scaffolding protein, and the NMDAR subunit, NR2A, accumulated in the amygdala. Coherently, contextual and cued fear memory was impaired. However, no change in hippocampal LTP was detected in this cdh1 mouse model [44].

### 4.2. APC/C-Cdh1 and Long-Term Depression

Huang et al. (2015) reported that APC/C-Cdh1 is involved in the regulation of long-term depression (LTD) [18]. They created a forebrain-specific conditional knockout of cdh1 and showed that metabotropic glutamate receptor (mGluR)-dependent LTD is impaired in the hippocampus in this mouse model. In hippocampus CA1 layer, it was shown that the induction of mGluR-dependent LTD is dysregulated, but not NMDAR-dependent LTD. 

**Figure 3:**
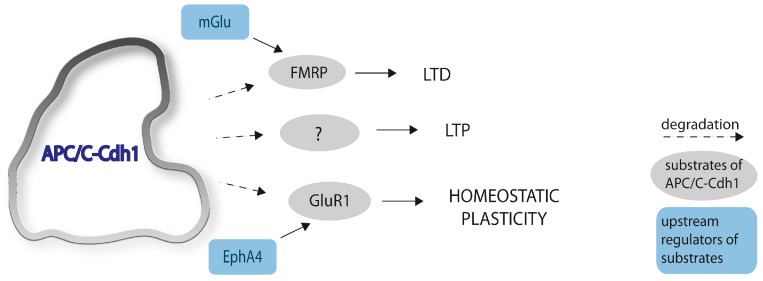
APC/C-Cdh1 regulates synaptic plasticity. The ubiquitin ligase APC/C-Cdh1 degrades FMRP and GluR1, thereby regulating LTD and homeostatic plasticity. To date, no direct APC/C targets have been identified that regulate LTP (potential unknown substrates indicated by ‘?’), but strong evidence suggests direct or indirect regulation of LTP the protein complex.

This study also reports that the fragile X syndrome protein (FMRP), which governs the mGluR-dependent LTD, is a substrate of APC/C-Cdh1. A major feature of the fragile X syndrome is exaggerated mGluR-dependent LTD. Surprisingly, mGluR-LTD is regulated by cdh1 in the cytoplasm rather than in the nucleus [18] (Figure 3).

Table 2 summarizes cdh1 knockout models that are discussed in this review. Many of these mouse models show alterations in the nervous system, which highlights the important role APC/C plays. 

## 5. APC/C and Its Emerging Role in Alzheimer’s Disease

The main pathological hallmarks of AD are the deposition of senile plaques and the formation of neurofibrillary tangles (NFTs). The plaques consist mainly of the toxic peptide Aβ, and NFTs are generated by the hyperphosphorylation of the microtubule-associated protein tau, forming aggregates. These pathophysiological characteristics are related to several other events that have been described in the disorder, such as oxidative stress, ectopic cell cycle re-entry, impaired LTP, excitotoxicity, and dysregulations of various signaling pathways. Finally, this results in neurodegeneration in AD brains. 

In this review, we propose the hypothesis that some of these disease-related events may be linked to the dysregulation of APC/C and its substrates. We recently showed in our laboratory that Aβ causes a proteasome-dependent degradation of cdh1. This was tested in vitro in neurons in primary culture and in vivo by microinjection in the rat hippocampus. Furthermore, APP/PS1 mice, (an experimental model of AD) have lower levels of cdh1 than age-matched wildtype mice [21]. These findings provide strong evidence for a direct involvement of APC/C-Cdh1 in the pathophysiology of AD. In the following section, we will discuss the dysregulation of substrates of the ubiquitin ligase in AD.

## 6. The Ectopic Cell Cycle in AD and Its Relation to APC/C-Cdh1

Once cells are committed to differentiate, they exit the cell cycle and remain in the G0 phase in a quiescent state. Terminally differentiated neurons in the adult brain are normally incapable of re-entering the cell cycle, partially due to an active degradation of cell cycle-related proteins. The re-entrance in an erroneous departure of G0 into the G1 and S phase was related to neurodegeneration [46]. The reactivation of an ectopic cell cycle in neurons has also been reported in AD. Elevated levels of cell cycle markers compared to age-matched controls appeared in neurons in the disorder [46,47,48,49]. Moreover, it was shown in these neurons that they have elevated markers of DNA replication, which indicates that they progress through the S-phase [50]. In cultured neurons in vitro, Aβ treatment leads to increased levels of cdk’s and cyclins, which causes them to pass through the S-phase, and they then enter into apoptosis before mitosis [51]. 

APC/C-Cdh1 targets cyclin B1 for degradation, which is a cell cycle protein, and it has been shown to accumulate in neurons in AD brains [2,52]. Maestre et al. (2008) showed that phosphorylation of cdh1 under excitotoxic conditions stabilizes cyclin B1 in neurons in primary culture, and thereby induces neuronal apoptosis [22]. They also reported that cdh1 is phosphorylated by cdk5, causing nuclear export and degradation of cdh1. Cdk5 is a non-traditional cdk, which is highly expressed in post mitotic neurons and is involved in the regulation of neuronal differentiation, migration, synapse formation, and synaptic plasticity [53]. The kinase activity of cdk5 is higher in AD than controls [24,25]. In cdk5^−/−^ knockout mice, cell cycle re-entry occurred in post-mitotic neurons, further supporting the evidence that cdk5- and cdh1-dependent downregulation of cyclin B1 acts as a cell cycle suppressor [26]. In turn, it has also been reported that APC/C-Cdh1 ubiquitinates cdk5 when it is transported to the cytoplasm, after initiation of the S phase [54]. 

These findings strongly suggest that APC/C-Cdh1 downregulation is involved in erroneous cell cycle re-entry in AD. 

## 7. Oxidative Stress in AD and Its Relation to APC/C-Cdh1

Perry and Smith’s pioneer work showed that there is oxidative damage in brains from AD patients [27]. Our group showed that Aβ interacts with heme groups in the mitochondrial membrane, causing interference with the normal electron flow. This results in the production of reactive oxygen species (ROS) and causes damage [55], which induces alterations in various signaling pathways in neurons that are related to oxidative stress and neurodegeneration. Among these, the downregulation of APC/C-Cdh1 in neurons induces a change in the oxidant and antioxidant homeostasis. 

Neurons are the cells in the brain which have the highest energy consumption but they have a comparatively low rate of glycolysis. They metabolize glucose through the pentose-phosphate pathway, which helps them to maintain their antioxidant status by regenerating reduced glutathione. Low levels of glycolysis are maintained by APC/C-Cdh1 in neurons, by targeting the enzyme 6-phosphofructo-2-kinase/fructose-2,6-bisphosphatase-3 (PFKFB3) for degradation. PFKFB3 generates fructose-2,6-bisoposphate, which is the most potent activator of the 6-phosphofructo-1-kinase (pfk1), one of the main regulators of glycolysis. 

The inhibition of cdh1 in neurons in culture leads to increased levels of PFKFB3, resulting in activation of glycolysis. Less glucose is used for the pentose-phosphate pathway and this causes oxidative stress and apoptosis [34]. Furthermore, an excitotoxic stimulus inhibits APC/C-Cdh1 activity, leading to an accumulation of PFKFB3, and thereby causes neurodegeneration [23]. In the AD mouse model TgCRND8, astrogliosis surrounding Aβ plaques with increased PFKFB3 activity was observed [56]. 

## 8. Excitotoxicity in AD and Its Relation to APC/C-Cdh1

Excitotoxicity, which refers to injuries caused by excessive concentration or prolonged exposure to the neurotransmitter glutamate, is described as a pathological phenomenon in various diseases, such as stroke, Huntington’s disease, and AD. Increased glutamate levels cause an overload of Ca^2+^ in neurons, which is a neurotoxic event that leads to the degradation of proteins, membranes and nucleic acids, induces oxidative stress, altered synaptic plasticity, apoptosis, and necrosis [57,58]. 

Glutamate is present in increased levels in the cerebrospinal fluid of AD patients, compared to healthy individuals of a similar age [59,60,61,62]. Currently, glutamatergic systems are one of the main therapeutic targets in AD treatment [63]. The toxic effect of Aβ on synapses can be partially ameliorated by the NMDA receptor antagonist memantine [64,65].

The increase in glutamate in AD was attributed to a failure in the glutamate recycling system. Thereby, glutamate cannot be properly taken up by astrocytes due to a decrease of the glutamate transporter GLT1, and the neurotransmitter thus remains in the synaptic cleft [66,67]. We found an additional mechanism of glutamate excitotoxicity in vitro and in vivo, in which the decrease of cdh1 caused by Aβ results in the stabilization of its substrate glutaminase, which is a main source of glutamate generation in neurons. We also observed increased glutaminase levels in the hippocampus in APP/PS1 mice compared to wildtype mice. These results suggest that excitotoxicity in AD might not only result from perturbations in the glutamate reuptake system, but also from increased glutamate production by glutaminase. 

Interestingly, a loss of glutaminase-positive neurons in late stages in AD has also been reported [68]. Moreover, glutaminase and glutamate immunoreactive neurons were correlated with the formation neurofibrillary tangles in AD [69]. Burbaeva et al. (2005) observed increased levels of glutaminase in the prefrontal cortex in AD brains [70]. The Aβ-induced decrease in cdh1 and the subsequent accumulation of glutaminase provide a possible explanation for these observations in AD. 

In a functional MRI study, it was shown that prodromal AD subjects have higher neural network activities than controls. It was suggested that this could be a compensatory mechanism due to a reduced number of neurons, or reflect a process of slow excitotoxicity [71]. Thereby, Aβ enhances neuronal sensitivity to glutamate, which could be mediated by several mechanisms, such as Aβ increasing Ca^2+^ influx and thereby causing an upregulation of signaling pathways that induce glutamate release, reduced glutamate uptake in the synaptic cleft, or increased activation of NMDA receptors [71,72]. We hypothesize that Aβ-induced glutaminase accumulation may be another molecular link between the pathogenic factors Aβ and slow excitotoxicity in AD.

## 9. LTP Impairment in AD and Its Relation to APC/C-Cdh1

Defects in LTP and LTD were shown in various cdh1 knockout models. Synaptic plasticity is often altered in neurobiological disease. Defects in LTP have been related to AD [73,74], and it has been reported that the AD-related mouse model APP/PS1 has decreased LTP compared to wild types (WT) [75]. We have observed that APP/PS1 mice have decreased levels of cdh1, which suggests that the lower APC/C-Cdh1 activity in these mice may contribute to the LTP deficiency [21]. 

Furthermore, EphA4 receptors, which are involved in excitatory neurotransmission, interact with APC/C-Cdh1 to stimulate the degradation of GluR1. Simon et al. (2009) reported that early changes in hippocampal Eph receptors precede the onset of memory decline in mouse models of AD. They found reduced EphA4 and EphB2 levels in the hippocampus, before the mice suffered memory impairments [76]. These reports suggest that the interaction of EphA4 and APC/C-Cdh1 signaling may be related to LTP deficiency in AD models. However, more research would be needed to clarify their role in LTP impairment in AD. 

## 10. Impaired Neurogenesis in AD and Its Relation to APC/C-Cdh1

A growing body of evidence indicates that neurogenesis is impaired in AD. It has been suggested that enhanced neurogenesis in the adult hippocampus represents an early critical event in the course of AD [77]. In APP/PS1 mice, an increased number in proliferating progenitors was detected using bromodeoxyuridine (BrdU) staining [78]. However, it has been shown in the dentate gyrus of AD brains that newly generated neurons do not become mature neurons even if neuroproliferation is increased [79].

Furthermore, Crews et al. (2011) showed that an alteration in cdk5 signaling is involved in defective neurogenesis in AD [80]. In an in vitro model of neuronal progenitor cells, which were exposed to Aβ and infected with a viral vector expressing p35, they observed neurite outgrowth and impaired maturation. This was reversed by inhibition of cdk5. Moreover, the inhibition of cdk5 rescued impaired neurogenesis in a transgenic mouse model of AD in vivo [81]. It has been reported that cdk5 phosphorylates cdh1 [22], thereby suggesting that APC/C-Cdh1 may be involved in the impaired neurogenesis in AD. 

Moreover, impaired neurogenesis in knockout models of cdh1 showed that the ubiquitin ligase has a major role in neuronal progenitors [10,41]. Furthermore, enhanced proliferation of neuronal progenitors together with a decrease in mature neurons, which has been observed in AD, is in line with the phenotype of cdh1 knockout models.

**Figure 4:**
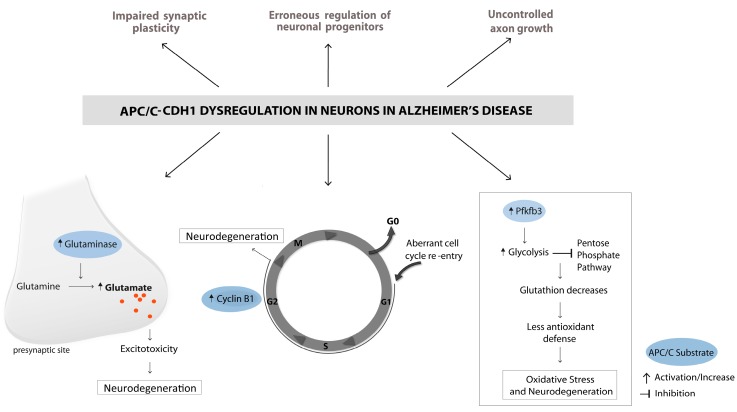
Dysregulation of APC/C-Cdh1 in Alzheimer’s Disease (AD) may affect several different functions in neurons. Pathways involving APC/C substrates that cause excitotoxicity, cell cycle re-entry, and oxidative stress have been related to neurodegeneration. Furthermore, APC/C inactivation in AD could potentially lead to dysregulation of synaptic plasticity, neurogenesis, and axon growth.

The involvement of APC/C in AD is summarized in Figure 4. 

## 11. Conclusions

The discovery of new substrates of APC/C is an active field of research [81], which is crucial for a better understanding of its function in health and disease. This approach depends on identifying potential recognition motifs based on their sequence, which can then be tested experimentally. A computational tool, a Group-based Prediction System for APC/C recognition motifs (GPS-ARM), was developed to identify potential substrates, which provides higher predictive power than the simple identification of D- and KEN-Boxes. In total, about 100 substrates have been experimentally identified, while computational analysis predicts several thousands of targets [82]. This suggests that many more substrates and functions of this ubiquitin ligase remain to be discovered. 

However, within the last two decades, substantial knowledge has been gained about the functions of APC/C-Cdh1 and APC/C-Cdc20 in neurons. To date, 15 substrates of this ubiquitin ligase have been identified that regulate important processes in the nervous system. It was shown that APC/C activity is essential for axon growth regulation [12,13]. Neurogenesis is another major process in the nervous system that underlies the control of APC/C [10], and it has been shown that the ubiquitin ligase and its targets control cortical size [10], prevent replicative stress [41], and regulate cell cycle exit [14] in neural progenitor cells. Furthermore, involvement of APC/C-Cdh1 in synaptic transmission and plasticity has been shown in Drosophila [28], *C.* elegans [42], and rodents [16]. Various studies in cdh1 knockout models show an essential role of APC/C-Cdh1 in LTP [43,44]. 

Dysregulation of APC/C activity seems to be involved in the pathophysiology of neurodegenerative diseases. As it controls a whole set of substrates, alterations of APC/C could affect many different proteins and their functions. Here, we have discussed several findings that relate APC/C-Cdh1 downregulation to AD. It has been shown that both glutamate excitotoxicity and Aβ oligomers decrease cdh1 protein levels, leading to inactivation of the ubiquitin ligase and to an accumulation of degradation targets [21]. It has been shown that cyclin B1, PFKFB3, and glutaminase accumulate in AD due to APC/C-Cdh1 inactivation [22,23,52,70]. We hypothesize that some other substrates of APC/C-Cdh1 may also accumulate in AD due to a downregulation of the ubiquitin ligase activity. Major functions, such as LTP and neurogenesis, that are affected in the disease [73,74,77] have been shown to be regulated by APC/C-Cdh1 [10,43,44]. Therefore, it would be interesting to investigate whether APC/C-Cdh1 dysfunction is responsible for alterations of these functions in AD. Further research is needed to fully elucidate its role in health and disease and may be a useful target for treatment of neurodegenerative diseases.

## Figures and Tables

**Table 1:** A list of Anaphase Promoting Complex/Cyclosome (APC/C) substrates that are directly involved in major functions in the central nervous system. APC/C-Cdh1 and APC/C-Cdc20 regulate neurogenesis, axon and dendrite growth, synaptic differentiation, synaptic regulation, cell cycle exit, neuronal survival and glucose metabolism in neurons.

UniProt Entry/Organism	Gene	Protein (APC/C Substrate)	Function	Motif	Coactivator	Reference
Q63689	*Neurod2*	Neurogenic differentiation factor 2	Regulation of presynaptic differentiation	D-box	cdc20	[20]
Rattus norvegicus						
Q9VM93	*syd-2*	Liprin-α	Regulation of synaptic size and activity/ γ-aminobutyric acid release at neuromuscular junction	D-box	cdh1/cdc20	[28,29]
Caenorhabditis elegans						
P19490	*Gria1*	Glutamate receptor 1 (GluR1)	Regulation of AMPA receptors in homeostatic plasticity	D-box	cdh1	[16]
Rattus norvegicus						
P41135	*Id1*	DNA-binding protein inhibitor ID-1	Involved in dendrite morphogenesis in neurons	D-box	cdh1/cdc2	[19]
Rattus norvegicus						
Q60665	*Skil*	Ski-like protein (SnoN)	Regulation of axonal morphogenesis	D-box	cdh1	[13,30]
Mus musculus						
Q9CUN6	*Smurf1*	E3 ubiquitin-protein ligase SMURF1	E3 Ubiquitin ligase that targets RhoA, regulates axon growth	D-box	cdh1	[31]
Mus musculus						
P41137	*Id2*	DNA-binding protein inhibitor 2 ID-2	Links axonal growth and cell cycle exit	D-box	cdh1	[14]
Rattus norvegicus						
Q06486	*Csnk1d*	Casein kinase I isoform delta	Regulation neurogenesis in cerebellum	D-box	cdh1	[32]
Rattus norvegicus						
O00716	*E2F3*	Transcription factor E2F3	Cell cycle exit and neuronal differentiation	D-box	cdh1	[33]
Homo sapiens						
P07818	*Ccnb1*	G2/mitotic-specific cyclin-B1	Maintains cell cycle exit and promotes neuronal survival	D-box	cdh1	[2,22]
Rattus norvegicus						
P07953	*Pfkfb3*	6-phosphofructo-2-kinase/fructose-2,6-bisphosphatase 3 (PFKFB3)	Regulation of glycolytic pathway in neurons, oxidative stress	KEN box	cdh1	[23,34]
Rattus norvegicus						
P13264	*Gls*	Glutaminase kidney isoform, mitochondrial	Regulates levels of neurotransmitter glutamate in neurons	KEN box	cdh1	[21,35]
Rattus norvegicus						
P35922	*Fmr1*	Synaptic functional regulator FMR1 (FMRP)	Drives mGluR-dependent synaptic plasticity	D-box	cdh1	[18]
Mus musculus						
Q8K0X8	*Fez1*	Fasciculation and elongation protein zeta-1 (FEZ1)	Dendrite growth in hippocampus	D-box	cdc20	[36]
Mus musculus						
Q8NEM0	*MCPH1*	Microcephalin MCPH1 (isoform B)	Cell cycle protein; homolog of a causative gene for autosomal recessive primary microcephaly in humans	D-box	cdh1	[37]
Homo sapiens						

**Table 2:** Summary of cdh1 knockout mouse models and their phenotype. Several impaired functions related to the nervous system have been observed in various cdh1 knockout mouse models.

Cdh1 knock-out *(Fzr1* gene)	Description	Phenotype defetcs	Reference
Gene-trap (gt) construct, inserted into intron 5 of *Fzr1*, generating a dysfunctional allele of *Fzr1*	Homozygous mice Cdh1^gt/gt^ (intercrossed heterozygous mice Cdh1^gt^)	Early embryonic lethality (died at ~E9.5), replicative senescence, premature fibroblasts	[17]
	Heterozygous Cdh1^gt^ (50% cdh1 reduction)	Defects in hippocampal late phase LTP, deficiency in contextual fear-conditioning	[17]
Two loxP sites eliminate exons 2 and 3 from the *Frz1* gene, cre recombinase expressed under the Sox2 promoter	Conditional cdh1 knockout mice (embryo restricted knockout)	Loss of genomic stability, increased susceptibility to spontaneous tumours	[45]
		Replicative stress, p53-mediated apoptotic death, alterations in neurogenesis resembling microcephaly	[10]
Two loxP sites eliminate exons 2 and 3 from the *Frz1* gene, cre recombinase expressed under nestin regulatory sequences	Conditional cdh1 knockout mice (knockout restricted to the developing nervous system)	Hypoplastic brain and hydrocephalus	[41]
Two loxP sites eliminate exons 2 and 3 from the *Frz1* gene, cre recombinase expressed under CaMKII promoter	Conditional cdh1 knockout mice (knockout restricted to excitatory neurons in the hippocampus and forebrain)	Impaired memory, impaired LTP in amygdala	[44]
Two loxP sites eliminate exons 2 and 3 from the *Frz1* gene, cre recombinase exressed under enolase promoter	Conditional cdh1 knockout mice (knockout restricted to neuronal expression from the beginning of development)	Impaired behavioral flexibility and extinction of previously consolidated memories, impaired LTP in hippocampal slices	[43]
Two loxP sites eliminate exons 2 and 3 from the *Frz1* gene, cre recombinase expressed under the control of the forebrain-specific driver Emx	Conditional cdh1 knockout mice (knockout in neocortical and hippocampal excitatory neurons but not GABAergic interneurons)	Profoundly impaired induction of mGluR-dependent LTD in the hippocampus	[18]
